# Expression and characterization of a potential exopolysaccharide from a newly isolated halophilic thermotolerant bacteria *Halomonas nitroreducens* strain WB1

**DOI:** 10.7717/peerj.4684

**Published:** 2018-04-24

**Authors:** Arpitha Chikkanna, Devanita Ghosh, Abhinoy Kishore

**Affiliations:** 1 Laboratory of Biogeochem-mystery, Centre for Earth Sciences, Indian Institute of Science, Bangalore, Karnataka, India; 2 Center for Nanoscience and Engineering, Indian Institute of Science, Bangalore, Karnataka, India

**Keywords:** Halomonas, Exopolysaccharide, Microbial phylogenetics, Halophilic thermophilic bacteria, Biopolymers

## Abstract

The halophilic bacterial strain WB1 isolated from a hydrothermal vent was taxonomically characterized using multiple proxies, as *Halomonas nitroreducens* strain WB1. When grown on malt extract/yeast extract (MY) medium, it produced large quantities of exopolysaccharide (EPS). The polymer was synthesized at a higher rate during the log and early stationary phases. The anionic polysaccharide is primarily composed of glucose, mannose, and galactose. The studied EPS was highly viscous and had pseudoplastic nature. The EPS was found to be a mixture of three polysaccharides under FT-IR, which makes it less labile to environmental diagenesis. It also has emulsifying and antioxidant activity along with the binding capacity to heavy metals. The EPS has unique and interesting physical and chemical properties, which are different from earlier reported exo-polysaccharides produced by different bacterial genus. This suggests that the extreme geological niches like hypersaline, hyperthermal, hypothermal, and oligophilic environments, which are not well studied so far, can offer extensive and potential resources for medical, biotechnological and industrial applications. The study clearly showed that the thermal springs from the temperate region can be a potent source of many such industrially important microbial genera and need further detailed studies to be carried out.

## Introduction

High molecular weight polysaccharides produced and released by different microbial groups are well known as exo-polysaccharides (EPSs) (Sutherland, 1990; Kumar, Mody & Jha, 2007). These complex molecules play a wide range of functions in the microbes such as binding to the substratum, migration and protection from desiccation, adherence of predators and antimicrobial agents released by the other organisms (Gerba & Bitton, 1984; Xu et al., 2010).

In industries, EPSs are very significant due to the diverse and complex structures of homo or hetero-polysaccharides with organic/inorganic substituent; they are widely used in food, pharmaceutical, paper, textile, paint, and the petroleum industry (Sutherland, 1998, 1999). However, EPSs have drawn more interest in the biotechnology industry because of their wide use in pharmacology, medicines, and cosmetics. Because of their efficiency, versatility, and biodegradability, EPSs are more preferred over natural or synthetic polymers in the biotechnology field (Mata et al., 2006; Quesada, Béjar & Calvo, 1993; Sutherland, 1999). Microbes producing EPSs can be found in diverse and extreme environments; however, such exopolysaccharide (EPS)-producing bacterial genera growing in extreme conditions (e.g., thermophilic, halophilic, psychrophilic) produce EPSs that are stable at these conditions. In high salt concentrations, unusually diverse halophilic bacterial populations can be found. These taxonomically diverse halophiles can also have diverse functional roles; for e.g., sulphur oxidation, denitrification, phosphate solubilization, cellulose degradation and production of EPS with antimicrobial, antiviral and antioxidant properties (Li et al., 2014). Such halophilic EPS with highly viscous and pseudoplastic nature can act as a salt-tolerant surfactant and have wide use in high salt oil deposits (Arias et al., 2003). They are widely used as viscosifier jellifying agents and/or emulsifying agents and also as metal binders (Quesada et al., 2004; Arias et al., 2003; Mata et al., 2006).

The Bakreshwar hot springs in West Bengal are of great ecological significance, but, are very less studied. This unique site could be the habitat of many ecologically significant microorganisms. Since these hot springs have an extreme thermal and saline condition, the authors hypothesized that this pristine niche might inhabit many un-identified strains which can have high biotechnological potential. The present study thus aims to (1) isolate and identify indigenous thermo-halophilic bacterial strains from the hot springs, (2) isolate EPSs produced by the thermo-halophilic bacterial strains to cope with the stressed environment, (3) determine chemical and physical characterization of the EPSs to understand its ecological and biotechnological significance.

## Geological Significance of the Sampling Site

**Figure 1:**
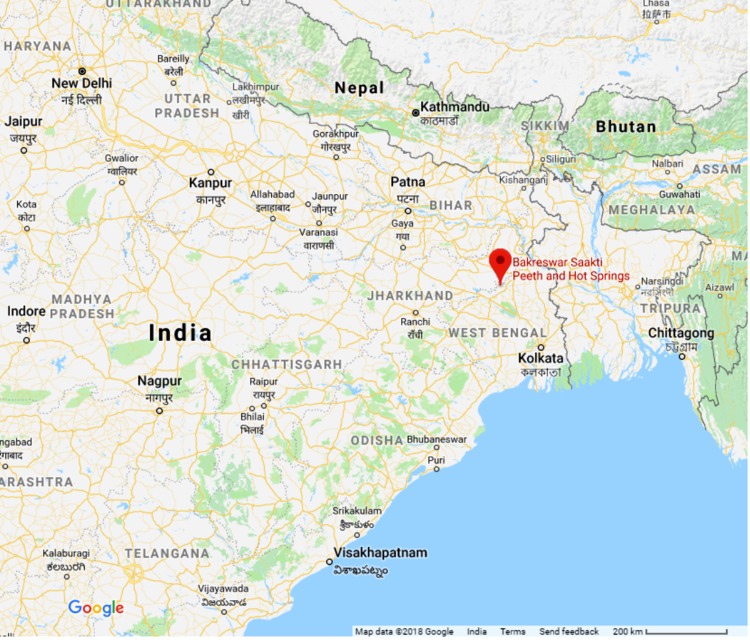
Location of Bakreshwar hot springs. Map data @2018 Google.

The Bakreshwar hot springs (23°52′48″N; 87°22′40″E; Fig. 1) are located in Birbhum district of the state West Bengal, India with a total of seven hot springs. The area comprises of Chhotanagpur gneiss, with intrusive dolerite and amphibolite dykes (Mukhopadhyay & Sarolkar, 2012). The temperature of these springs varies from 33 to 70 °C, with profuse hot water and gaseous discharge containing 0.31% to 1.33% He (Mukhopadhyay & Sarolkar, 2012).

## Materials and Methods

### Sampling

Most hot spring tanks showed a thin layered microbial mat at the photic zone; however, to avoid the algal deposition, we selected the hot spring named Agnikunda. Six sampling stations were identified surrounding Agnikunda, among the seven springs of Bakreshwar in December 2015. Each of these tanks was sampled thrice. We collected 3–5 g of surface soil sample submerged at a depth of 0.5 m below the water table and a water sample from the same depth. Water samples were collected before soil samples in order to reduce any particle dispersion. Physicochemical properties of the soil and water such as pH, Eh, temperature, and dissolved oxygen, were measured in situ and the samples were stored in sterile containers and kept at 4 °C until carried to the laboratory. The soil pH was measured by making 2:5 ratio of soil: water suspensions, using a glass electrode (Digital pH meter; Systronics, Ahmedabad, India). The electrical conductance of soil-saturation extract was also measured (Sartorius PY-Y12, Göttingen, Germany).

### Isolation of EPS producing bacteria

The collected soil samples were used as primary inoculums to isolate the bacterial strains. 1 g of the soil was dissolved in 10 ml of 5% NaCl solution and was further diluted upto 10^7^. The suspension was spread on saline Malt extract/yeast extract (MY) agar plates (1% dextrose, 0.5% peptone, 0.3% yeast extract, 0.3% malt extract, 5% NaCl, and 1.5% agar; Moraine & Rogovin, 1966). The plates were prepared in triplicates for each sample and were then incubated in inverted condition for 48 h at 60 °C. High incubation temperature was selected based on the water temperature of the spring. This also allowed only indigenous thermophilic bacterial strains from the hot spring to grow, inhibited the growth of any mesophilic contaminant; expelling the chances of handling error. Of the MY agar plates prepared from the six station, the sample from the first station didn’t show any growth. In the other 15 plates, very small colonies were observed. Of which only five different bacterial strains showed a slimy surface (an indication of EPS production) and were isolated and sub-cultured in the MY agar plates, and re-incubated at 60 °C for one week. One of the isolates showed a high production rate of a thick gel-like substance which got deposited on the lid of the Petri plate as incubated inversely. This strain named as WB-1 was then further characterized by the following downstream analyses.

### Taxonomic characterization of EPS producing bacteria

Polyphasic taxonomic approaches were taken to identify the bacterial strain. The oxidase activity of WB-1 oxidizing bacterial isolates was detected using 1% *p*-aminodimethylaniline oxalate and the catalase activity was detected using 3% (v/v) H_2_O_2_ solution. Utilization of 30 different carbon sources was tested using KB009 HiCarbo™ Kit (HiMedia, Mumbai, India) following the manufacturer’s instructions.

**Figure 2:**
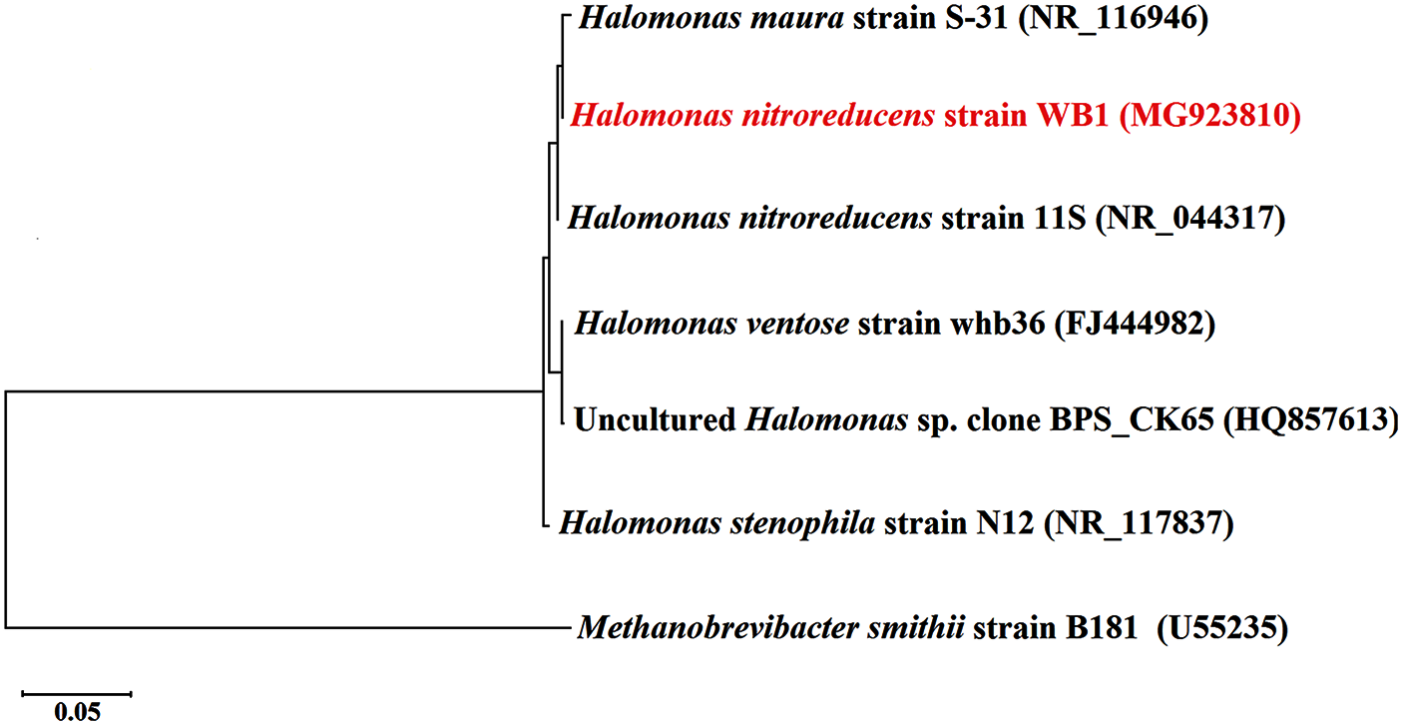
Maximum likelihood phylogenetic tree of 16S rDNA sequence of the bacterial isolate (in red). The 16S rDNA of *Methanobrevibacter smithii* B181 (Accession No. U55235) was used as outgroup to root the tree. [Scale bar indicates 0.05 substitution per site].

For molecular characterization of WB-1, its genomic DNA was extracted (Sambrook & Russel, 2001) and the polymerase chain reaction (PCR) amplification of its partial 16S rDNA was targeted using previously published eubacterial primers Fc27 (5′-AGAGTTTGATCCTGGCTCAG-3′) and Rc1492 (5′-TACGGCTACCTTGTTACGACTT-3′; Lane, 1991). Each of these PCR reactions consisted of 0.5 μl DNA Dream Taq polymerase (5 U/μl) (Fermentas), 5.0 μl 10× Dream Taq buffer, 5.0 μl dNTPs (final concentration 0.2 mM), 5.0 μl MgCl_2_ (final concentration 2.0 mM), 0.5 μl of each primers (final concentration 5 μM), 0.5 μl (∼20 ng) DNA template, 0.5 μl BSA (1 mg/ml), and nuclease-free water to make a final volume of 50 μl. The reaction conditions are as follows: initial denaturation at 95 °C for 10 min, 36 cycles of 95 °C for 1 min, 55 °C for 1 min, 72 °C for 3 min 30 sec, and a final extension at 72 °C for 10 min. The PCR reactions were performed in triplicates pooled down and purified using the Gel Purification Kit (Qiagen, Hilden, Germany) as per the manufacturer’s instructions. The purified product was then sequenced in both directions using the same primer in an ABI Prism 3730 Genetic Analyzer based on Big Dye Terminator chemistry (Applied Biosystems, California, United States). The sequence was checked in Bellerophon (Huber, Faulkner & Hugenholtz, 2004) for chimera and had been submitted to GenBank database accession number MG923810. It was then compared against nucleotide databases (GenBank/EMBL/DDBJ) using the blastn tool (Camacho et al., 2008). The top five 16S rDNA sequences in the match result were used for phylogenetic representation (Fig. 2). The phylogenetic representation of the identified strain was done using Maximum likelihood (ML; Saitou & Nei, 1987) method based on Kimura 2 parameter model (Kimura, 1980) for constructing the tree. The phylogenetic tree was rooted using the 16S rDNA sequence of *Methanobrevibacter smithii* strain B181 (accession number U55235) as outgroup.

### Growth of the strain and EPS production

The isolated bacterial strain was maintained in saline MY media (mentioned above). In the same medium, various growth conditions were tested, such as, NaCl concentration (2.5%, 5%, 7.5%, and 10% wt/vol), growth temperature (25, 35, 40, 50, and 60 °C) and pH (5.5, 6, 6.5, 7, 7.5, and 8). The production of EPS was carried out in a 1 l flask containing 300 ml of saline (5% NaCl) MY media (pH 7.5). The flasks were incubated on a rotary shaker incubator at speed 110 rpm and temp 60 °C for one week.

### Isolation and extraction of EPS

The bacterial cells were harvested from 2 l of saline MY medium, using extended high-speed centrifugation, 8,000 rpm for 30 mins to the supernatant, 2 vol of chilled isopropanol (99.5%; Sigma-Aldrich, Bengaluru, India) was added and kept at 4 °C for 24 h for precipitation. It was then centrifuged at 10,000 rpm for 30 min in a cold centrifuge and the precipitated white material was slowly dried at 50 °C in a hot air oven.

### Characterization of physical properties of EPS

The dried EPS was dissolved in sterile mili Q water (0.5% w/v) and the rheological property was measured at a shear rate 0–100 s^−1^ at 25 °C in a controlled-stress Rheolyst AR1000N (TA Instruments, Bengaluru, India). To measure the emulsifying property of the EPS, a previously described method was used (Cooper & Goldenberg, 1987; Llamas et al., 2006). Triton X-100 and Tween 80 (Sigma-Aldrich, Bengaluru, India) were used as control surfactants. Equal volume of each of these solutions in mili Q water (0.5% w/v) were mixed with hydrophobic substances: sunflower oil, mustard oil, olive oil (commercial products), octane, Vaseline oil, hexadecane, and toluene (Sigma-Aldrich, Bengaluru, India), mixed vigorously and kept at 4 °C undisturbed for 24 h. The emulsifying property is described as the percentage (%) of the volume of the emulsion.

Metal-binding property of the EPS was characterized by equilibrium dialysis experiments as described previously (Geddie & Sutherland, 1993; Morillo et al., 2006). For the dialysis, 200 ml 0.1 mM solution of the cations (Cadmium, Cobalt, Copper, Lead, Nickel, and Zinc) was prepared in mili Q water. A mixed solution (0.1 mM) of all the six selected cations was also used to study the selective adsorption of metal to the polymer. Dialysis tubing (Sigma D9777, 12,000 Da and above) was pre-treated by washing under running distilled water for 3–4 h and then treating it with a mixture of 0.3% (w/v) sodium sulphide and 1 mM EDTA at 80 °C for 60 sec followed by mili Q water at 60 °C for 2 min and acidification with 0.2% H_2_SO_4_. The acid was then removed by washing the tube with hot mili Q water. Five ml of EPS solution in mili Q water (0.5% w/v) was placed into the dialysis tube and was released into the flask containing 200 ml of the respective cation solution. The flask was then gently shaken at 100 rpm for 24 h at room temperature. The cation solution was sampled at 0 h and 24 h and immediately acidified with 1% nitric acid solution. The cations were quantified using an atomic absorption spectrophotometer (PerkinElmer 5100, Waltham, MA, United States).

The metal uptake *Q* in the solution was determined as follows:

$$Q = {{V({C_i}-{C_f})} \over m}$$

Where, *C_i_* is the metal concentration in solution of volume (*V*) at 0 h, *C_f_* is the equilibrium concentration of the metal in solution (at 24 h), and *m* is the mass of the EPS (Morillo et al., 2006).

### Characterization of chemical properties of the EPS

The total spectrophotometric quantification of the following was done: total carbohydrate (Dubois et al., 1956), total protein content (Bradford, 1976), acetyl residues (McComb & McCready, 1957), pyruvate (Sloneker & Orentas, 1962), and hexosamines (Johnson, 1971). Ash content was determined gravimetrically after baking the EPS at 550 °C for 6 h. To analyze the net ionic charge, the purified EPS was loaded on an anion exchange cartridge: quaternary methyl ammonium column (1.5 m × 20 cm; Waters, Millipore). The flow rate in the column of 0.05 M NH_4_HCO_3_ (pH 8.0) was 2 ml/min followed by a linear gradient of 0.05–2 M NaCl in 0.05 M NH_4_HCO_3_ (pH 8.0). The monitoring of EPS was done at 210 nm (UV). Fractions of 5 ml were collected to determine their total saccharine composition and molecular mass as described earlier Mata et al. (2006).

### Antioxidant properties of the EPS

The hydroxyl radical scavenging property of EPS was detected using Fenton reaction in comparison to ascorbic acid (Zhang et al., 2013). Each reaction mixture consisted of EPS sample of various concentrations (0.1, 0.25, 0.5, 1, 2, 3, 4, 5 mg/ml) in mili Q water, 2.0 ml of FeSO_4_ (0.5 mM), 1.0 ml of brilliant green (0.435 mM) and 1.5 ml of H_2_O_2_ (3% w/v). The absorbance was measured spectrophotometrically at 624 nm. The scavenging activity (SA) is reported as:

$${\rm{SA}}\% {\rm{ }} = {\rm{ }}\left[ {\left({{\rm{AS }}-{\rm{ A0}}} \right)/\left({{\rm{A }}-{\rm{ A0}}} \right)} \right]{\rm{ }} \times {\rm{ }}100$$

Where, AS is the absorbance of the sample, A0 is the absorbance of the control and A is the absorbance of the Fenton reaction mixture at 624 nm.

The scavenging of 2,2-diphenyl-1-picrylhydrazyl (DPPH) radicals by the EPS was quantified using the previously published method (Kao & Chen, 2006) with some modifications (Zhang et al., 2013). The SA% is reported as mentioned above.

## Results

### Biochemical and molecular identification

The isolated EPS producing bacteria was identified by various biochemical and molecular characterizations. The biochemical tests including C-source utilization for 30 different C-sources has been detailed in Table 1. The 16S rDNA of the strain WB1, showed 99% identity with *Halomonas nitroreducens* strain 11S (Accession No. NR_044317), at nucleotide level, and inferred as *H. nitroreducens* strain WB1. The phylogenetic identification of the strain showed that it belongs to the bacterial phylum *Proteobacteria*, class *Gammaproteobacteria*, order *Oceanospirillales*, family *Halomonadaceae*, genus *Halomonas* (Fig. 2).

**Table 1:** Biochemical characterization of *H. nitroreducens* strain WB1.

Strain Name	Gram charater	Motility	Catalase activity	Oxidase activity	Starch hydrolase activity	Esculin hydrolysis	Nitrate reductase activity	Carbohydrate utilization																																	
								Lactose	Xylose	Maltose	Fructose	Dextrose	Galactose	Raffinose	Trehalose	Melibiose	Sucrose	L-Arabinose	Mannose	Innulin	Sodium gluconate	Glycerol	Salicin	Dulcitol	Inositol	Sorbitol	Mannitol	Adonitol	Arabitol	Erythritol	α-methyl D glucosyl	Rhamnose	Cellobiose	Melezitose	α-methyl D manoside	Xylitol	ONPG	D-Arabinose	Citrate utilization	Malonate utilization	Sorbose
WB1	−	−	+	+	+	−	+	+	−	+	+	+	+	+	−	−	−	+	+	+	−	+	−	−	−	+	+	−	+	−	−	−	+	−	−	−	−	+	+	−	−

**Notes:**

+ indicates positive result/isolate is able to utilize the C source.

− indicates negative result/isolate is unable to utilize the C source.

### Bacterial growth kinetics vs EPS production

**Figure 3:**
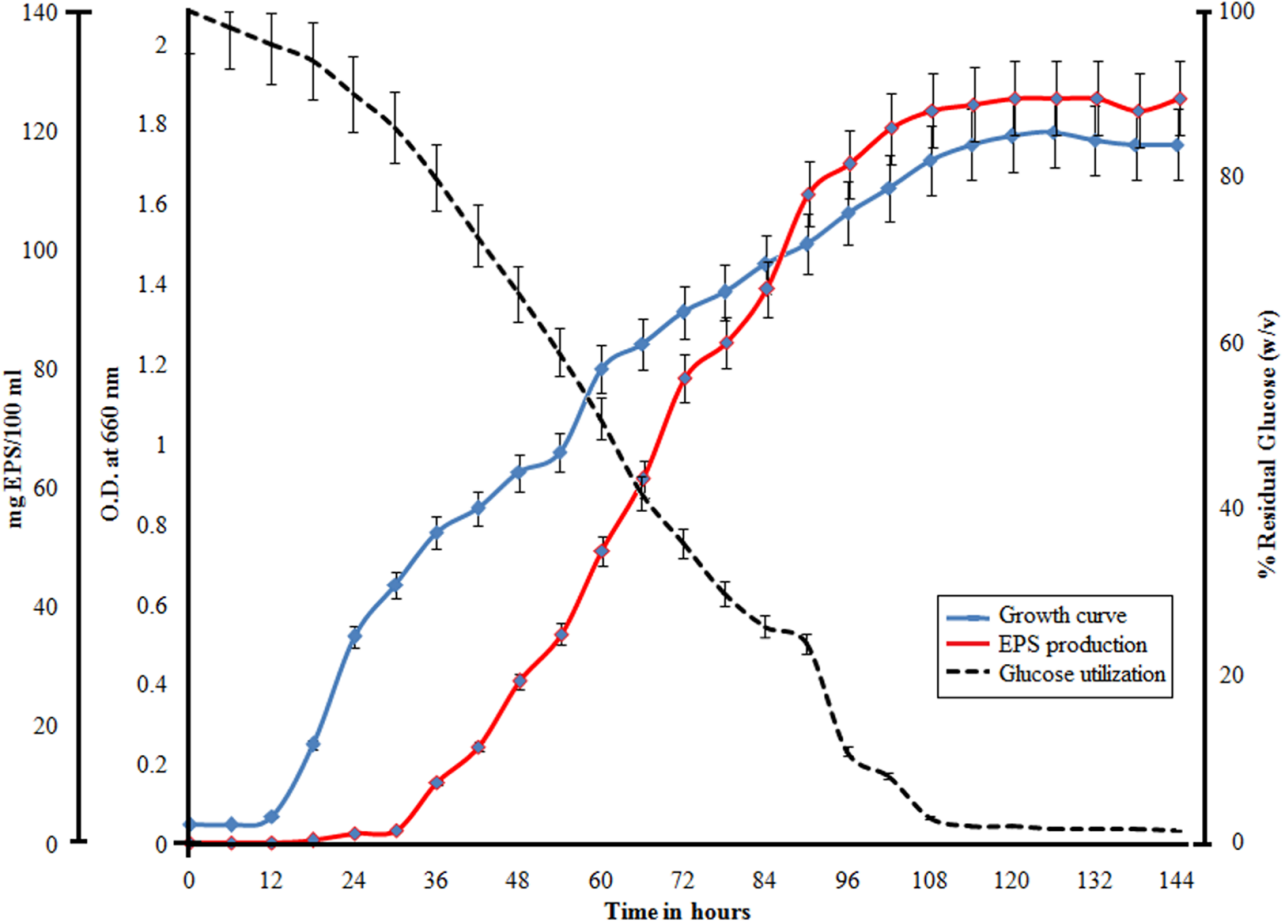
Demonstration of bacterial growth along with utilization of glucose and EPS production by *H. nitroreducens* strain WB1. All the variables are plotted with respect to time (*X*-axis). The blue line indicates growth curve, the red line indicates EPS production and the dotted line indicates the utilization of glucose. 100% glucose corresponds to 10 g/L of glucose.

EPS production by the bacterial strain in the saline MF media was monitored for seven days at 60 °C at 110 rpm. The 1% dextrose present in the media sustained and increased the biomass production and EPS secretion. The EPS secretion reaches a peak during the late log phase and continues in the stationary phase (Fig. 3). During the death phase the residual glucose levels increased from 10.5% to 14%.

### Physical properties of the EPS

**Figure 4:**
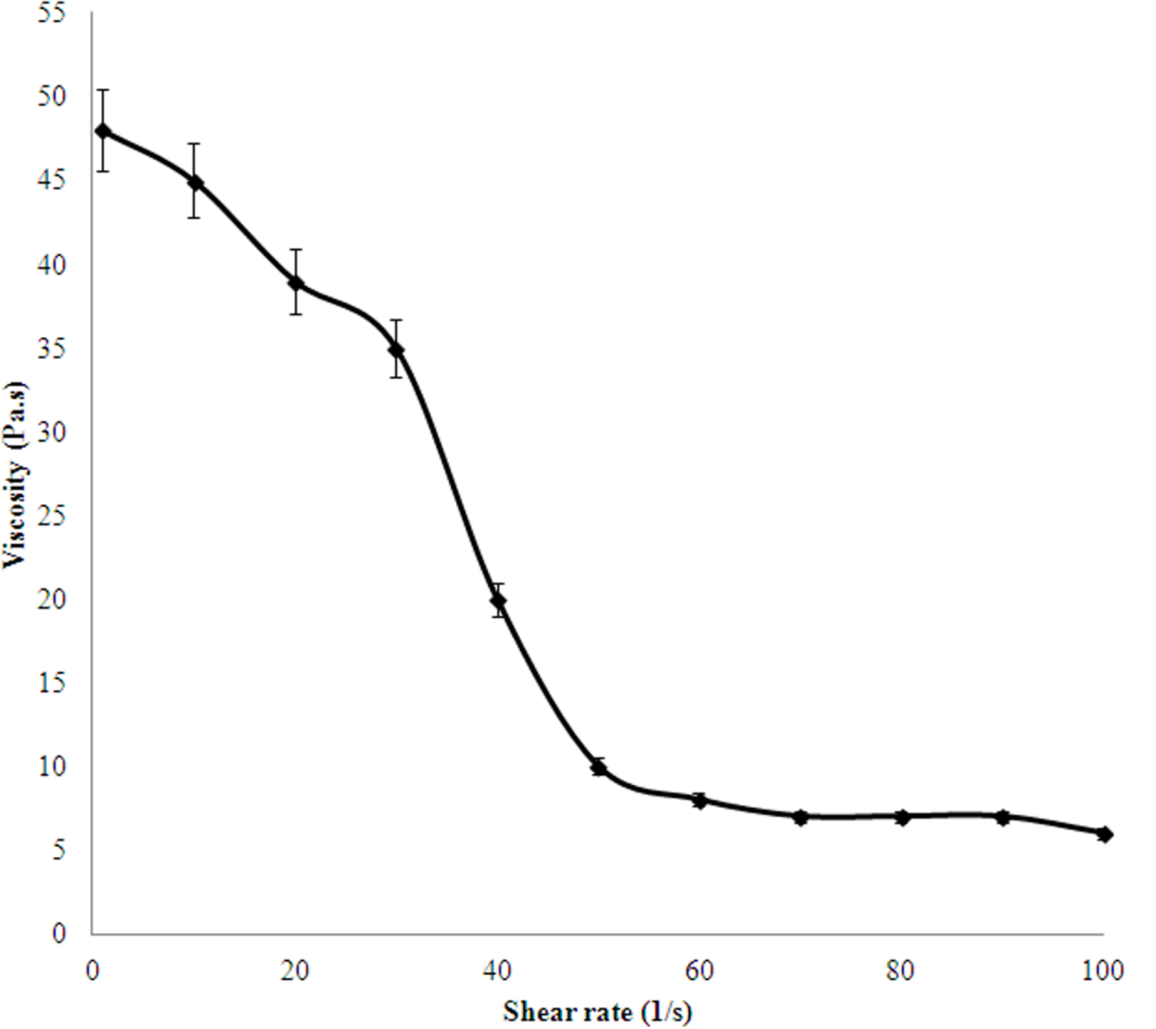
Viscosity index of 0.5% (w/v) EPS produced by *H. nitroreducens* strain WB1.

The rheological property of the EPS studied has been plotted in Fig. 4. It shows that the EPS is highly viscous and the viscosity decreased with increase in shear rate, indicating a pseudo-plastic nature of the polymer.

The emulsifying activity of the EPS secreted by the studied bacterial strain was measured with respect to two other surfactants Tween X-100 and Tween 80, and detailed in Table 2. The EPS stabilized the oil and water mixture with greater efficiency than Tween 80 and Tween 100. The highest emulsifying activity was detected with sunflower oil.

**Table 2:** Emulsifying activities of the EPS produced by *H. nitroreducens* strain WB1 in comparison to chemical surfactants.

	Sunflower oil	Mustard oil	Olive oil	Octane	Vaseline oil	Hexadecane	Toluene
WB-1	85 ± 2.5	68 ± 2.5	68.4 ± 0.5	62.04 ± 0.78	65 ± 0.55	56.3 ± 1.2	56.7 ± 1.2
Triton X-100	65.3 ± 1.5	66 ± 2	66.1 ± 1.2	62.3 ± 0.45	60.2 ± 0.2	55.9 ± 0.33	53.4 ± 0.72
Tween 80	63.2 ± 1.5	59.3 ± 0.5	65.6 ± 1.33	61.8 ± 1.2	61 ± 0.52	55 ± 0.5	52.7 ± 0.8

**Note:**

About 0.5% (w/v) of EPS or the chemical surfactants (Triton X-100 and Tween 80) were used as emulsifiers. The total volume of the oil-water emulsion observed after 24 h, has been expressed as the percentage. Each value represents the average of triplicate data measurements and hence the standard deviation is given.

**Figure 5:**
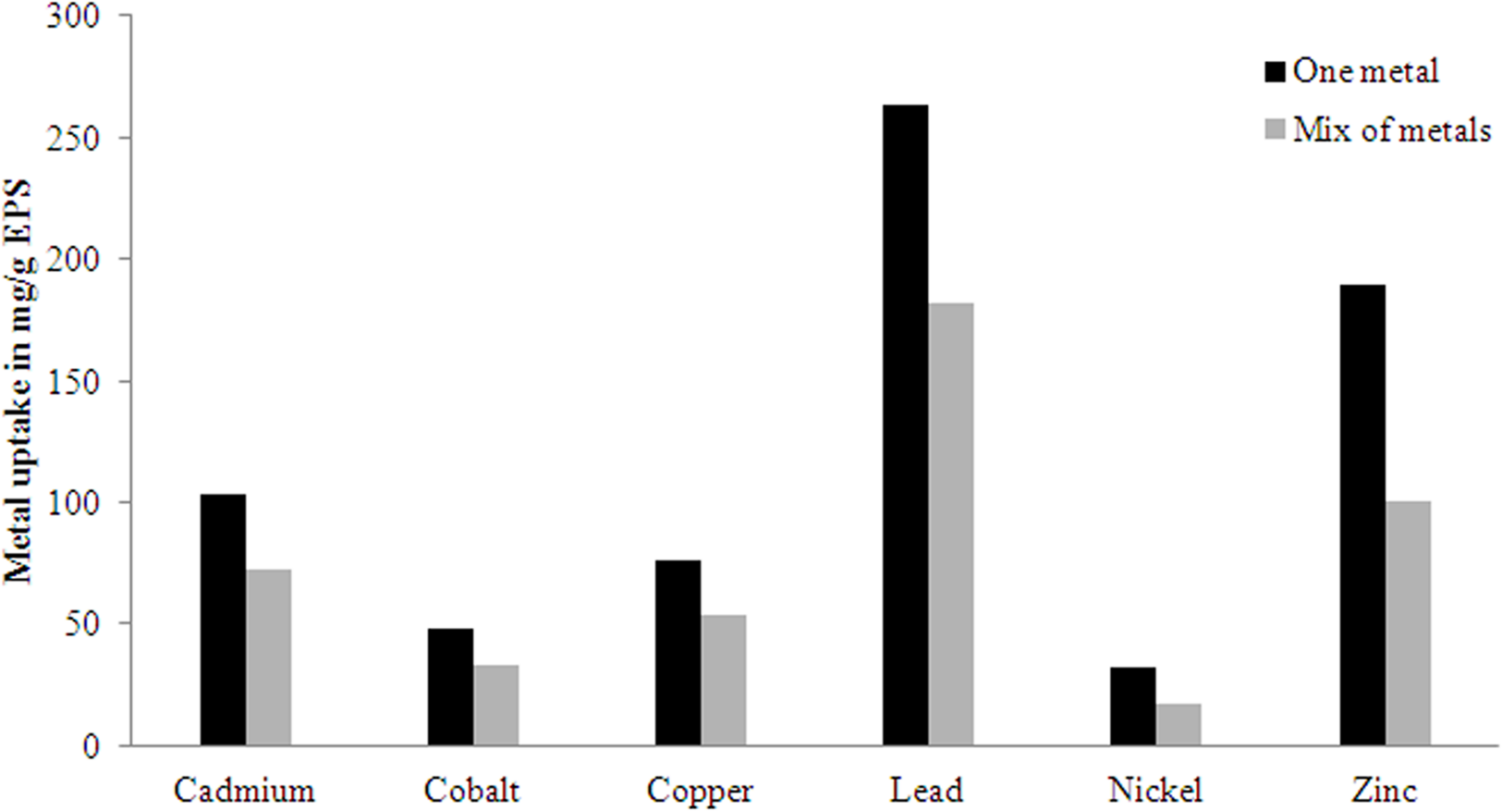
Metal uptake (Q) by the EPS produced by *H. nitroreducens* strain WB1 in one metal and mixed sorption experiment.

Among the six metals used to test the metal binding efficiency of the EPS produced by the studied bacterial strain, the affinity for Pb was the highest (Fig. 5). In the mixed metal solution, the partial affinity of EPS for Pb was 182 mg/g, however in uni-metal solution it was 263 mg/g. The binding affinity followed the order Pb>Zn>Cd>Cu>Co>Ni.

### Chemical properties of the EPS

Under optimum conditions the EPS produced by the strain WB1, composed of 68.9% (w/w) carbohydrates, 2.2% (w/w) proteins, 0.7% (w/w) acetyls, 7.2% (w/w) pyruvic acid, and 1.9% (w/w) hexosamines. All the values represent the average of triplicates of the quantification.

**Figure 6:**
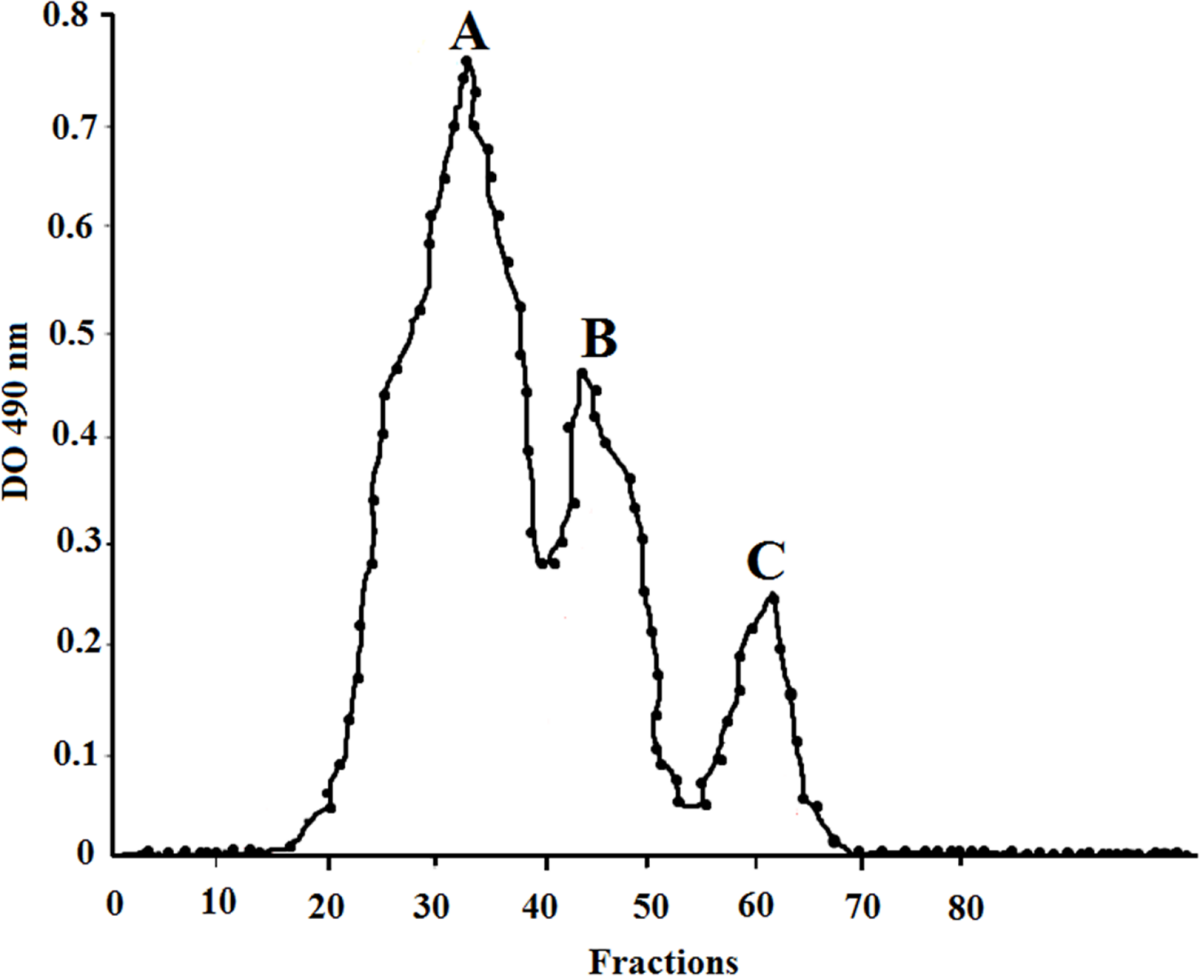
Anion-exchange chromatogram of EPS produced by *H. nitroreducens* strain WB1, showing two high molecular weight peaks (A) and (B), and one low molecular weight peak (C). Each peak is characterized and described in Table 3.

Three chromatographic peaks were detected when loaded on an anion-exchange cartridge, inferring that the polysaccharide from *H. nitroreducens* strain WB1 contains at least three species (Fig. 6). The size distribution of these peaks showed peak A of 5.2 × 10^6^ Da, peak B 3.0 × 10^4^ Da and peak C of 1.3 × 10^3^ Da. The monosaccharide composition of each peak of the studied EPS is detailed in Table 3. The primary components of high molecular weight fraction (peak A) were glucose (28%) and mannose (64%) with small quantities of galactose (6%). The main components of peak B were glucose (18.5%), mannose (44%) and low quantity of rhamnose and arabinose. The low molecular weight fraction (peak C) consisted of glucose (19%), mannose (56.5%), galactose (14.2%) and low quantities of galactouronic acid. It showed no peaks in a cation-exchange CM Sep-Pak cartridge.

**Table 3:** Three polysaccharide peaks were observed during anion exchange chromatography of the extracted EPS (see Fig. 6)

		Fractions of anion exchange chromatogram		
		Peak A	Peak B	Peak C
	Molecular weight	5.2 × 10^6^ Da	3.0 × 10^4^ Da	1.3 × 10^3^ Da
Monosaccharide (%, w/w)	Glucose	28	18.5	19
	Mannose	64	44	56.5
	Galactose	6	ND	14.2
	Rhamnose	ND	2	ND
	Arabinose	ND	1.5	ND
	Xylose	Traces	Traces	ND
	Fructose	ND	ND	Traces
	Galactouronic acid	ND	ND	1.5

**Notes:**

This table represents the molecular masses and monosaccharide composition of the polysaccharide represented by each peak of the EPS produced by *H. nitroreducens* strain WB1.

ND is not detected.

### Antioxidant properties of the EPS

#### Hydroxyl radical scavenging activity

**Figure 7:**
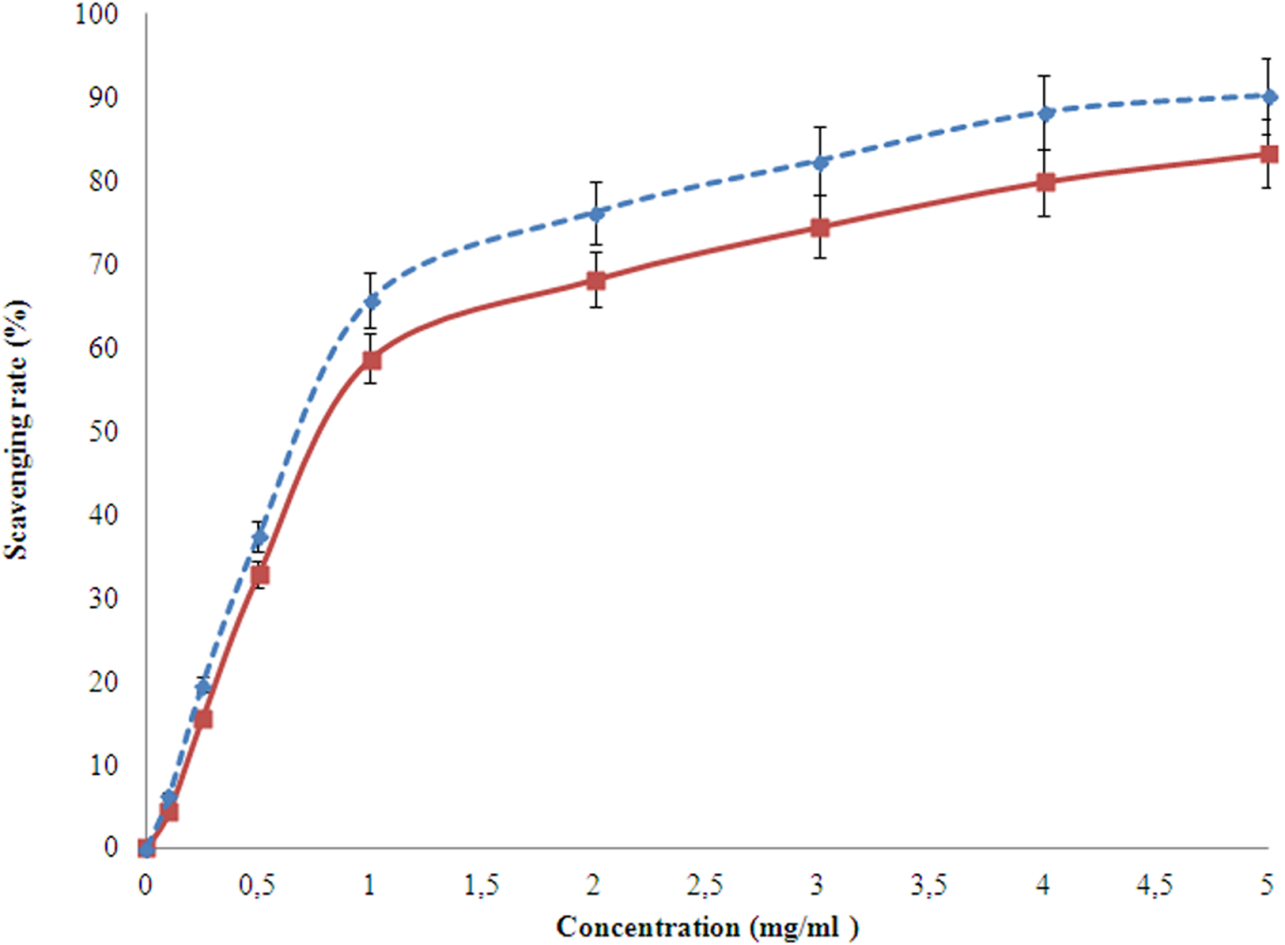
Hydroxyl radicals scavenging activity of the EPS (red) produced by *H. nitroreducens* strain WB1 with respect to that of ascorbic acid (blue).

Hydroxyl radicals are the most reactive species causing severe damage to the living cells and biomolecules. The ability of the studied EPS to scavenge these radicals is detected using the Fenton reaction with respect to the scavenging property of ascorbic acid (Fig. 7). The scavenging activity had a linear relation with that of concentration. The EPS of the organism in this study can scavenge from 0.1 to 4.8 mg/l. The scavenging activity was higher at higher concentrations (Fig. 7) than ascorbic acid.

#### DPPH radical scavenging activity

**Figure 8:**
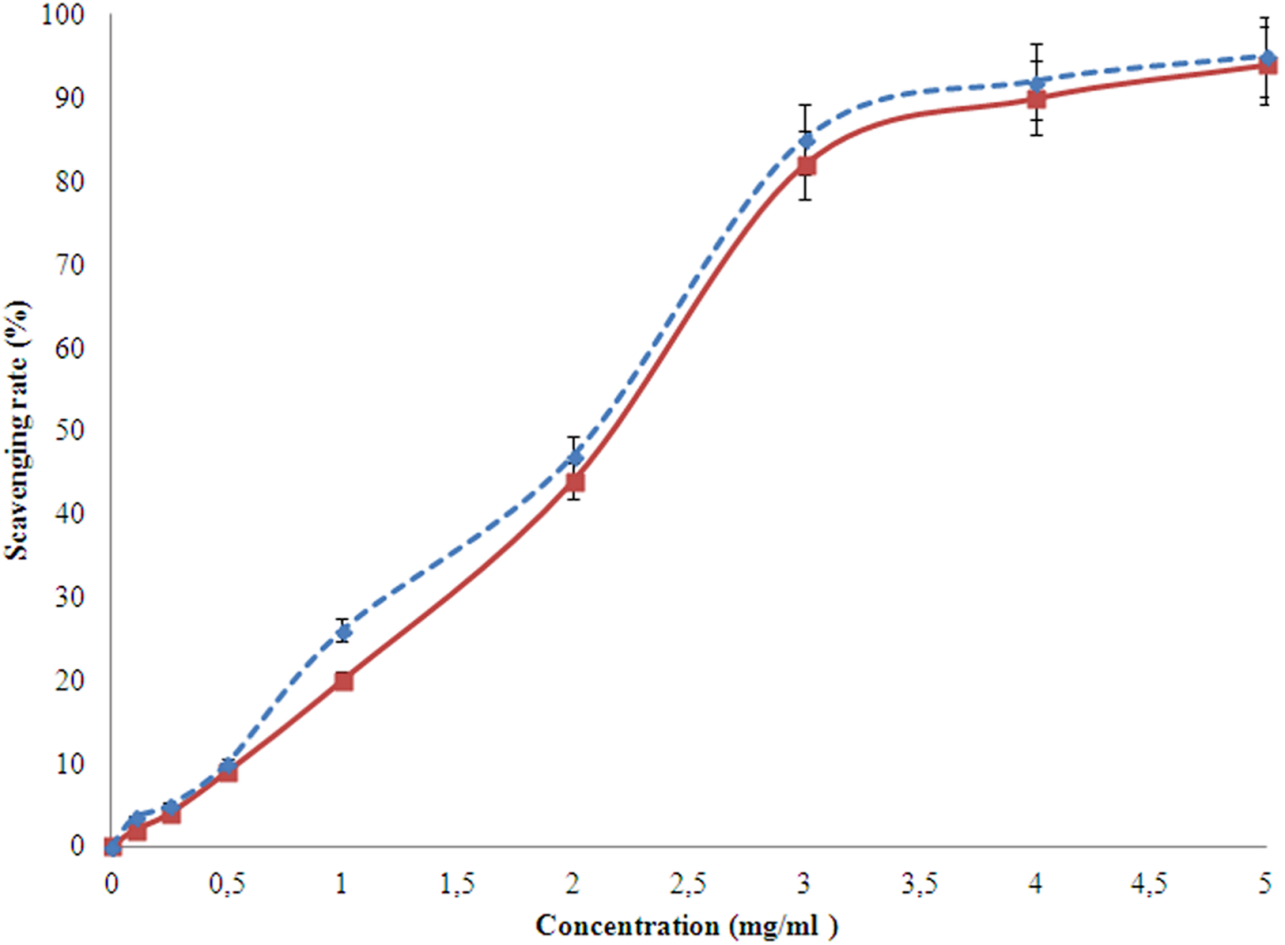
DPPH scavenging activity of the EPS (red) produced by *H. nitroreducens* strain WB1 with respect to that of ascorbic acid (blue).

The scavenging activity of the studied EPS was measured spectrophotometrically, with respect to ascorbic acid. Alike hydroxyl ion, the DPPH ion scavenging also takes place higher at higher concentrations. However, after 1 mg/l concentration of EPS, the rate of increase in scavenging activity reduced down (Fig. 8). At the concentration of 5.0 mg/ml, the EPS and ascorbic acid showed 83.3% and 90.2% of DPPH radical scavenging activity, respectively.

## Discussion

The water of the hot spring Agnikunda is HCO_3_—Cl type, with high HCO_3_, Cl^−^ and Na^+^ ions (Mukhopadhyay & Sarolkar, 2012). The enrichment of heavier stable isotopes indicates a deep circulation of meteoric waters in the geothermal system (Bandopadhyay, 1991). The bacterial strain isolated from this saline environment was identified by its 16S rDNA characterization as a halophilic bacteria *H. nitroreducens* strain WB1. The optimum temperature for growth and EPS production was at 60 °C and hence it is also a thermophilic strain. This non-motile, Gram negative bacterial strain is an aerobic nitrite reducer and is able to utilize a large number of monosaccharide as C source (Table 1). The property of this bacterial strain to grow at high temperature, utilizing an array of C sources, makes it industrially significant; also the salinity makes it easier to maintain its pure culture. The EPS production was monitored with respect to growth and the glucose consumption which was measured at laboratory conditions (Fig. 3). The production of EPS starts in the log phase of growth and the rate reaches to maximum after three days. The production also occurs in the early stationary phase of growth and slows down and stops by late stationary phase (Fig. 3). Such growth patterns and EPS production had also been reported in earlier reports (Mata et al., 2008; Llamas et al., 2006).

The aqueous solution of the EPS was very viscous which can be an influence of the molecular mass and various groups (discussed further). The flow curve (Fig. 4) shows a pseudo-plastic nature of the EPS. The emulsifying activity is described in Table 2. The EPS efficiently stabilizes the mixtures of different oils and water. The hydrophobic phases were vegetable oil or mineral oil or a hydrocarbon. The emulsifying activity of the studied EPS was better in comparison to the synthetic surfactants used as control in this study. The emulsion produced by the EPS synthesized by *H. nitroreducens* strain WB1 formed uniform micro-droplets which were stable, giving it a smooth consistency. Thereby, the EPS can be used in oil and food industries as an eco-friendly emulsifier (Desai & Banat, 1997; Banat, Makkar & Cameotra, 2000; Mata et al., 2008). Such bio-surfactants, because of their bio-compatibility, non-toxicity and efficiency at low concentrations have many industrial applications (Sutherland et al., 2004; Uzoigwe et al., 2015).

The metal binding ability of the studied EPS shows that the bacteria *H. nitroreducens* strain WB1 can be used efficiently in waste-water treatment plant for floccules formation and in polluted environments. The metal binding activity is mostly driven by bioadsorption and might be forming ionic bonds (Valls & de Lorenzo, 2002). The EPS has high affinity for Pb similar to the earlier reported bacterial EPSs from *Alteromonadaceae* family (Arias et al., 2003; Mata et al., 2006, 2008). Such anionic EPSs have high affinity for the large cations (Geddie & Sutherland, 1993). Such property of the EPS can be largely utilized in developing bio-compatible and environment-friendly ion-exchange resins. The remediation of a heavy metal pollutant from refining ores, mines, sludge, paints, pesticides, and other industries can be done using such binding and adsorption properties of complex EPSs (Ansari, Masood & Malik, 2011).

The EPS reported in this study is primarily composed of carbohydrates, proteins, acetyls, pyruvic acid, and hexosamines alike most microbial EPS reported from halophiles (Sutherland, 1990; Arias et al., 2003; Quesada et al., 2004; Mata et al., 2006, 2008). Three chromatographic peaks were detected when loaded on an anion-exchange cartridge, inferring that the polysaccharide from *H. nitroreducens* strain WB1 contains at least three species of polysaccharides (Fig. 6). The size distribution of these peaks showed, peak A of 5.2 × 10^6^ Da, peak B 3.0 × 10^4^ Da and peak C of 1.3 × 10^3^ Da. The monosaccharide composition of each peak of the studied EPS is detailed in Table 3. The primary components of high molecular weight fraction (peak A) were glucose (28%) and mannose (64%) with small quantities of galactose (6%). The main components of peak B were glucose (18.5%), mannose (44%) and low quantity of rhamnose and arabinose. The low molecular weight fraction (peak C) consisted of glucose (19%), mannose (56.5%), galactose (14.2%) and low quantities of galactouronic acid. It showed no peaks in a cation-exchange CM Sep-Pak cartridge.

The EPSs described so far from halophilic bacteria such as *Idiomarina fontislapidosi* and *I. ramblicola* or lactic-acid bacteria *Streptococcus thermophilus*, are either formed of one or two fractions (Ricciardi et al., 2002; Arias et al., 2003; Vaningelgem et al., 2004; Mata et al., 2006, 2008). Those EPSs were of molecular mass 2 × 10^4^–4 × 10^6^ Da. One of the important features of the EPS synthesized by *H. nitroreducens* strain WB1 generated three peaks (A, B and C; Fig. 6) corresponding to three fractions. This infers the production of three different molecular-mass of the EPS. This unique property makes the EPS less labile to degradation by common bacteria and fungus (Bhaskar & Bhosle, 2005). All the three fractions were mainly composed of glucose and mannose with low quantities of rhamnose, arabinose, galactose, and galactouronic acid. The physical properties of an EPS such as its rheology, emulsifying efficiency and metal chelating property can be greatly influenced by its molecular mass distribution. Here, the unique properties of the EPS make it an industrially important product as it will have greater bio-compatibility and will also have longer shelf life (Bhaskar & Bhosle, 2005; Sutherland et al., 2004).

The DPPH free stable radical has a wavelength of 517 nm and is able to scavenge an electron from any antioxidant to form a stable diamagnetic molecule (Fujito, 1981; Soare et al., 1997). The EPS under this study shows high rate of scavenging activity till 1 mg/ml after which it reduces. Such a property had also been reported for other EPSs (Zhang et al., 2013).

The hydroxyl radical (OH) on the other hand are highly reactive species of hydroxide ion (OH^−^). Although it is produced by the immune system in higher animals due to infections, it can react with several biomolecules and cause many diseases (Lipinski, 2011). The hydroxyl radical scavenging property of the studied EPS with respect to ascorbic acid was determined using Fenton’s reaction. The scavenging activity increased with increase in concentration of the EPS for up to 3 mg/ml, where the scavenging activity of EPS (83%) was relatively similar to that of ascorbic acid (85%). The result show the EPS can be potentially used as scavenging biomolecules commercially as encouraged by earlier groups (Liu et al., 2012; Zhang et al., 2013). With the non-toxic, biocompatible antioxidant property, the reported EPS provides a large range of its application in food preservation and cosmetic industry.

## Conclusion

Industrially important biopolymers from extremophilic organisms not only have robust properties but also are low cost to produce. This is because the extreme conditions of incubation, which forbid any common airborne contaminant to grow. Although EPSs are secreted by many aquatic microorganisms to get protection from predators, but for extremophilic organisms, they also act as a protectant from environmental conditions. Therefore, such robust polymers have high potential in many industries. In this study, a thermo-halophilic bacteria *H. nitroreducens* strain WB1 was isolated based on enrichment culture technique. The bacterial strain was able to produce high quantities of EPS at stressful physicochemical conditions, leading to exclude the chances of cross contamination of the bulk culture by common mesophilic bacteria at ease. The various chemical and physical properties of the EPS were studied and it was found that it is relatively unique. The EPS is a mixture of three polymers and this unique property makes it relatively less labile to degradation than the EPSs reported so far. It has a pseudo-plastic nature with emulsifying, anti-oxidation and metal binding properties, making it industrially potent for many applications like in food, paints, drugs and so on.

## Supplemental Information

10.7717/peerj.4684/supp-1 Supplemental Information 1 Sequence data.

## Additional Information and Declarations

### Data Availability

The following information was supplied regarding data availability: GenBank: MG923810 Sequence data is also available as a Supplementary File.
